# Genotype-Phenotype Correlations in Monogenic Parkinson Disease: A Review on Clinical and Molecular Findings

**DOI:** 10.3389/fneur.2021.648588

**Published:** 2021-09-22

**Authors:** Daniele Guadagnolo, Maria Piane, Maria Rosaria Torrisi, Antonio Pizzuti, Simona Petrucci

**Affiliations:** ^1^Department of Experimental Medicine, Policlinico Umberto i Hospital, Sapienza University of Rome, Rome, Italy; ^2^Department of Clinical and Molecular Medicine, Sapienza University of Rome, Rome, Italy; ^3^Medical Genetics and Advanced Cell Diagnostics Unit, S. Andrea University Hospital, Rome, Italy

**Keywords:** Parkinson's disease, phenotype, monogenic, early onset parkinsonism, Juvenile parkinsonism

## Abstract

Parkinson disease (PD) is a complex neurodegenerative disorder, usually with multifactorial etiology. It is characterized by prominent movement disorders and non-motor symptoms. Movement disorders commonly include bradykinesia, rigidity, and resting tremor. Non-motor symptoms can include behavior disorders, sleep disturbances, hyposmia, cognitive impairment, and depression. A fraction of PD cases instead is due to Parkinsonian conditions with Mendelian inheritance. The study of the genetic causes of these phenotypes has shed light onto common pathogenetic mechanisms underlying Parkinsonian conditions. Monogenic Parkinsonisms can present autosomal dominant, autosomal recessive, or even X-linked inheritance patterns. Clinical presentations vary from forms indistinguishable from idiopathic PD to severe childhood-onset conditions with other neurological signs. We provided a comprehensive description of each condition, discussing current knowledge on genotype-phenotype correlations. Despite the broad clinical spectrum and the many genes involved, the phenotype appears to be related to the disrupted cell function and inheritance pattern, and several assumptions about genotype-phenotype correlations can be made. The interest in these assumptions is not merely speculative, in the light of novel promising targeted therapies currently under development.

## Introduction

Parkinson disease (PD) is a complex, progressive, neurodegenerative disorder with worldwide incidence of 5–35 in 100,000 cases per year and prevalence reaching 2–4% at the age of 85. PD prevalence is expected to double in the next two decades because of population aging. Mortality is higher in patients with a more than 10-year-long history of disease (1). Pathological hallmarks of the condition include Lewy bodies, Lewy neurites, and loss of dopaminergic neurons of the *substantia nigra* (SN) *pars compacta* (SNpc). However, PD neuropathology is pleomorphic, as Lewy bodies are absent in some monogenic forms of PD (2). Clinical manifestations include motor signs (resting tremor, rigidity, bradykinesia, and postural instability) and non-motor features such as hyposmia, constipation, mood disorders, and rapid eye movement sleep behavior disorder (RBD), often preceding the motor signs. In later stages, cognitive decline and autonomic dysfunction may appear. The mean age of onset is in the sixth decade of life, ranging from <40 to more than 80 years. Early-onset PD (EOPD) is commonly defined as an age of onset under 45 years, while juvenile Parkinsonism (JOPD) refers to those cases with onset within 21 years (3).

A PD case is defined as familial or sporadic, according to the presence or absence of a clear family history. Approximately 5–10% can be classified as familial (4).

Most PD cases have a multifactorial etiology, resulting from the combined effects of environmental and genetic factors, while about 5–10% are caused by pathogenic variants in single genes. Monogenic forms of PD are more frequent in EOPD patients, being more than 10% of cases with onset before 45 years and more than 40% in those with onset before 30 years (5). Familial and monogenic PD must not be regarded as synonymous because many familial cases do not have a Mendelian transmission model. To date, more than 20 genes whose mutations cause autosomal dominant (AD), autosomal recessive (AR), and X-linked Parkinsonisms are known, with related phenotypes ranging from idiopathic PD-like (iPD-like) to young-onset Parkinsonisms, either pure or complicated by atypical motor and non-motor features (6). This review will focus on monogenic conditions with Parkinsonian signs as the prominent feature. Parkinsonisms presenting as an iPD-like condition will be classified as “typical PD.” Cases presenting complex phenotypes, featuring prominent additional neurological signs, such as dementia, spasticity, dystonia, and/or abnormal ocular movements, will be classified as “atypical PD.” We will discuss the clinical presentation and genetic cause of each condition, with insights into underlying molecular pathology, to provide genotype–phenotype correlations. Genetic variants will be identified with the most widely used traditional literature nomenclature. The phenotypes will be presented and discussed individually, based on transmission patterns and clinical features. A comprehensive overview of the discussed conditions is provided in Table 1.

**Table 1:** Genes causing Parkinsonian conditions and related phenotypes.

**Gene**	**MIM**	**Function**	**Disease onset**	**Phenotype**	**Additional features**	**Neuropathology^#^**
**Autosomal dominant, confirmed**						
*LRRK2*	609007	Lysosomal	Late/variable	Typical	–	±Lewy body; ±tau
*VPS35*	601501	Vesicular trafficking	Late	Typical	–	–
*SNCA*	163890	Unknown	Late/early^*^	Atypical/typical^*^	D; PS; PYR; MYO	Lewy body
*GCH1*	600225	Monoamine synthesis	Variable	Typical	–	Lewy body
*ATXN2*	601517	mRNA transport/regulation	Early	Typical	–	Lewy body
**Autosomal dominant, to be confirmed**						
*HTRA2*	610297	Mitochondrial	Late	Typical	–	Unknown
*GIGYF2*	607688	Possibly IGF-1 signaling	Late	Typical	–	Unknown
*UCHL1*	613643	Ubiquitin-proteasome	Late	Typical	–	Unknown
*EIF4G1*	614251	Protein synthesis	Late	Typical	–	Unknown
*CHCHD2*	616244	Mitochondrial	Variable	Typical	–	Lewy body, tau
*DNAJC13*	614334	Vesicular formation	Late	Typical	–	Lewy body, tau
*TMEM230*	617919	Vesicular trafficking	Late	Typical	–	Lewy body, tau
*RIC3*	610509	Acetylcholine receptor assembly/expression	Variable	Typical	–	Unknown
**Risk factor for PD**						
*GBA*	606463	Lysosomal	Late/variable	Atypical/typical	D	Lewy body
**Autosomal recessive**						
*PRKN*	602544	Mitochondrial	Early/juvenile	Typical	–	–
*PINK1*	608309	Mitochondrial	Early	Typical	–	± Lewy body
*DJ-1*	602533	Mitochondrial	Early	Typical/typical	–	± Lewy Body
*VPS13C*	608879	Mitochondrial/vesicular trafficking	Early	Atypical	D; PYR	Lewy body
*PTRHD1*	617342	Ubiquitin-proteasome	Early	Atypical	ID; PYR; PSY	Unknown
*PODXL*	602632	Neurite outgrowth	Juvenile	Typical	–	Unknown
*DNAJC6*	608375	Synaptic endocytosis	Juvenile	Atypical	D; PSY; SZ; PYR	Unknown
*SYNJ1*	604297	Synaptic endocytosis	Juvenile	Atypical	D; SZ; DYS; OM	Unknown
*ATP13A2*	610513	Lysosomal	Juvenile	Atypical	D; PYR; SVGP; mini-MYO	Lipofuscin deposits
*PLA2G6*	603604	Membrane homeostasis/mitochondrial	Juvenile	Atypical	D; PYR; ATX; PSY; OM	Lewy body
*FBX7*	605648	Ubiquitin-proteasome/mitochondrial	Juvenile	Atypical	PYR; PSY	Unknown
**X-linked**						
*RAB39B*	300774	Vesicular trafficking	Early in males	Atypical in males	ID; MC	Lewy body

*D, dementia; PSY, psychiatric disorders; PYR, pyramidal signs; MYO, myoclonus; ID, intellectual disability; SZ, seizures; OM, ocular motion disorders; DYS, dystonia; SVGP, supranuclear vertical gaze palsy; ATX, ataxia*.

* *= in peculiar gene alterations*;

# *= beyond loss of dopamine neurons*.

### Autosomal Dominant PD

Autosomal dominant PD (AD PD) includes different forms of Parkinsonisms that share many peculiarities such as incomplete penetrance, a mean age of onset during the fifth decade or later, and a good response to dopaminergic treatment. However, additional neurological signs, when present, distinguish iPD-like conditions from atypical Parkinsonism. Pathogenic variants in *LRRK2* and *VPS35* are usually related to Parkinsonisms resembling typical PD, while alterations in *SNCA* are more frequently found in atypical forms. However, phenotypes may overlap.

#### Idiopathic PD-Like

##### LRRK2

Pathogenic variants in the *LRRK2* gene (leucine-rich repeat kinase 2, MIM^*^609007), also known as *Dardarin*, are the most commonly known causes of AD PD, accounting for 5% of familial and 1% of sporadic cases (PARK8, MIM#607060) (7). They were identified more than 15 years ago by two independent groups in two unrelated families, with late-onset Parkinsonism resembling iPD (8, 9). Many rare *LRRK2* variants have been detected over the years but only six of them (p.R1441G/C/H, p.G2019S, p.Y1699C, and p.I2020T) are considered disease causing (8–13). Three additional variants (p.A211V, p.K544E, and p.T1410M), recently demonstrated to cause neurotoxicity, are waiting for confirmation (14). Two coding substitutions, p.G2385R and p.R1628P, mostly identified in Asian populations, act instead as genetic risk factors, each conferring a 2-fold risk of developing PD (15, 16). The p.G2019S variant is by far the most common, being detected in 4% of familial and 1% of sporadic PD cases worldwide (17). Its frequency is even higher among Mediterranean populations and some ethnic groups, including Ashkenazi Jews and North Africans, in which it is found in 23 and 37% of patients with familial PD, respectively (18). Conversely, p.G2019S is extremely rare in East Asia (17). The other known pathogenic *LRRK2* variants are very rare, with the exception of p.R1441G and p.R1441C, which are founder mutations in Basque and south Italian ethnicities, respectively (19, 20). The penetrance of the p.G2019S variant is incomplete and age-dependent, peaking at 42.5–74% at the age of ~80 years (17, 21), but varies among different ethnicities (22). Additional genetic variants, acting as single or cumulative risk factors, have been demonstrated to contribute to such variability (23, 24). Age-related penetrance has also been reported for the p.R1441G mutation, ranging from 13% at age 65 to 83% at age 80 (25). Less is known about the penetrance of the other pathogenic variants.

The *LRRK2*-related phenotype, closely resembling iPD, is characterized by a late-onset progressive Parkinsonism, with resting tremor as a common presenting feature, good response to levodopa therapy, and, usually, positive outcomes with deep brain stimulation (DBS) (17, 26). However, the mean age of onset is slightly lower in *LRRK2-*related cases than iPD, as patients with PD onset before 40 years are more common among the p.G2019S carriers (7), and differs among different populations, for example, being 10 years earlier among Tunisian carriers compared to Norwegian ones (22). Further features differentiating *LRRK2* p.G2019S-related Parkinsonism from iPD are the absence of gender differences (27), the slower progression for motor signs and cognitive impairment (28), and the more frequent occurrence of postural-instability-gait-difficulty (29). Typical iPD non-motor features, such as hyposmia, sleep, and cognitive and dysautonomic alterations, occur in *LRRK2* PD cases, but with less frequency (30). Dementia is also rarer in *LRRK2*-related Parkinsonism (17). When present, cognitive deterioration is usually milder and more slowly progressive than in iPD (31) and is characterized by better attention, executive function, and language (32). Pathogenic variants in *LRRK2* were found to be extremely rare in multiple system atrophy (MSA), progressive supranuclear palsy, and corticobasal degeneration (33–36).

Prodromal and premotor symptoms in *LRRK2* carriers are still poorly known. In pre-diagnostic PD phases, asymptomatic cases may present subtle motor alterations or isolated Parkinsonian signs, including decreased arm swing, gait asymmetry, and voice changes (37). Non-motor symptoms, such as constipation and urinary urgency, anxiety, daytime sleepiness or poorer performances in executive functioning, and subtle gait changes, are more frequent in asymptomatic *LRRK2* variant carriers than in heathy controls (7).

Brain imaging alterations have been found in *LRRK2* mutation carriers. Increased gray matter volume of different anatomical structures associated with motor loops has been documented in symptomatic and asymptomatic *LRRK2* carriers compared to controls. In contrast, a decreased basal ganglia gray matter volume has been found in *LRRK2* PD cases (38). Inversion recovery MRI sequences, assessing brain iron content, showed excessive iron deposition in the SN of brains from *LRRK2* carriers (39). Abnormal DAT-SPECT scans have been found in all *LRRK2* patients manifesting PD, as well as in some carriers showing prodromal signs and in a subgroup of non-manifesting carriers (40). Moreover, PET studies showed increased dopaminergic and cholinergic activity in *LRRK2* non-manifesting carriers compared to sporadic PD cases, possibly reflecting compensatory changes preceding the motor onset of PD (41, 42).

The neuropathology of *LRRK2*-related disease, mainly investigated in p.G201S carriers, is characterized by neuronal loss in the SNpc and *locus coeruleus* in all cases, with or without protein aggregation. Typical Lewy-type pathology and alpha-synuclein (α-syn) aggregates are present in 65–80% of *LRRK2* manifesting carriers, at a lower frequency than iPD cases (43). Tau inclusions, with variable distribution and severity, are also common, being found in about half the brains from patients with *LRRK2*- PD (43). a-syn aggregates prevail in p.G2019S carriers, while pure nigral degeneration has been described in about half of p.I2020T patients. Nevertheless, neuropathology can differ among relatives carrying the same pathogenic variant (44). Neuropathological and clinical features are strictly correlated, as Lewy-type pathology has been associated with the occurrence of non-motor symptoms, while pure neurodegeneration has been found in brains from patients with PD with only motor signs (43, 44).

More recently, genome-wide association studies (GWAS) confirmed the linkage of *LRRK2* locus variants to sporadic PD (45–47). Contrary to rare pathogenic mutations, these common variants confer modest risk for PD, suggesting a possible role of *LRRK2* in influencing iPD susceptibility (37).

*LRRK2* is a large and multifunctional protein with multiple domains for various protein–protein interactions and enzymatic serine–threonine kinase and GTPase activities. All the clearly pathogenic mutations cause a toxic gain-of-function. The increase in the *LRRK2* kinase activity mainly compromises neuronal vesicular trafficking, through an aberrant excessive phosphorylation of Rab GTPases (48). These findings have suggested that *LRRK2* kinase inhibitors might be a therapeutic target in *LRRK2*-related PD not only for monogenic *LRRK2*-related Parkinsonisms but also for the more common iPD (49).

##### VPS35

Late-onset PD with autosomal dominant inheritance (PARK17, MIM#614203) is also the phenotypic picture related to pathogenic variants in the *VPS35* gene (vacuolar protein sorting 35, MIM^*^601501). The phenotype is quite similar to iPD, mostly homogeneous and with a benign course. It may differ for an earlier age of onset (mean age at onset 50 years) (50). No atypical signs have been described in the *VPS35* p.D620N variant carriers since its discovery in two unrelated families with Swiss and Austrian origin, respectively (51, 52). Scarce cognitive and neuropsychiatric features, hyposmia in about 50% of patients, and excellent response to levodopa are reported (52, 53). Among these kindreds, the penetrance was high but incomplete (53). Beyond the p.D620N, identified in a few other familial or sporadic PD cases worldwide, no other proved pathogenic variants have been reported (54). The only patient who underwent neuropathology after death did not show signs of α-syn (55).

The *VPS35* protein is part of the retromer complex, involved in the neuronal vesicular recycling from endosomes to the trans-Golgi network. Altered *VPS35* is supposed to compromise the intracellular localization and stability of these organelles (56).

#### Atypical AD PD

##### SNCA

Pathogenic variants in *SNCA* (alpha-synuclein, MIM^*^163890), encoding α-syn, were identified 20 years ago as the first genetic cause of AD PD (PARK1, MIM#168601; PARK4, MIM#605543). In the large “Contursi kindred” the *SNCA* p.A53T mutation segregated with a PD and dementia with Lewy bodies phenotype (DLB, MIM#127750) (57, 58). Many other point mutations and whole gene multiplications have been detected in hundreds of patients with hereditary forms of autosomal dominant forms of PD, DLB, and other neurodegenerative conditions thus far (14). The identification of α-syn as a major component of Lewy bodies (LBs) and Lewy neurites (LNs), the neuropathological pathognomonic hallmarks of PD and DLB, confirmed the role of *SNCA* in the pathogenesis of PD. The detection of Lewy-type pathology in sporadic PD supported the involvement of α-syn in iPD, too (44, 59). Damaging mutations and whole gene multiplications favor α-syn aggregation with potential deleterious consequences at both the synaptic level and lysosomal/endosomal compartments, by a gain-of-function mechanism (60).

Duplication and triplications of an otherwise normal *SNCA* gene have been described in more than 100 patients (61). *SNCA* missense mutations (p.A53T, p.A30P, p.E46K, p.H50Q, and p.G51D) are instead very rare worldwide.

Many different PD phenotypes have been related to *SNCA* mutations, ranging from the more common late-onset Parkinsonism, either with or without non-motor symptoms, to the rarer early-onset aggressive diseases with atypical signs.

In whole-gene multiplications, the number of *SNCA* copies clearly correlates with the disease severity, supporting the notion of a “dosage effect” (62). Indeed, patients with four copies of *SNCA* (heterozygous triplication or homozygous duplication carriers) have a 10-year earlier age of onset, a more rapid progression and a more severe phenotype, often complicated by myoclonus, severe cognitive impairment, and psychiatric features, compared to heterozygous duplication carriers (61). Marked weight loss, dysautonomia, and fatigue can precede motor symptoms onset with death occurring within 7 years. Brain imaging reveals frontoparietal atrophy and a severe striatal dopaminergic deficit (63, 64). *SNCA* duplications cause a more variable phenotype, even within the same family, ranging from asymptomatic carriers to iPD-like or, more rarely, to severe forms resembling *SNCA* triplication carrier phenotypes (61, 65). The mean age of onset for *SNCA* duplication-related PD is 50 years. Non-motor symptoms are inconstantly present, and death occurs after 15 years from onset (66, 67). Atypical phenotypes have been described, including fronto-temporal dementia (FTD) with marked anxiety and obsessive–compulsive disorder, and a singular head shaking movement disorder (68, 69).

Compared to copy-gain variants, missense mutations cause more complex phenotypes with mutation-specific trends in clinical presentations (70). In most cases, despite similar ages of onset (average age 47.6 + 12.9 years), motor and non-motor features differ among patients with different specific mutations.

The p.A53T PD is characterized by marked intra-familial and inter-familial variability (67, 71). Penetrance is incomplete but high (80–90%). Parkinsonism resembles iPD, with a more aggressive and rapid course. Tremor is not common. Motor signs are initially L-dopa responsive but worsen early because of the occurrence of motor complications. Non-motor features, including hyposmia, orthostatic hypotension, RBD, and depression are inconstantly present. Myoclonic jerks and central hypoventilation have been reported. Cognitive impairment may vary, but dementia usually occurs within 5–7 years from disease onset (67, 72, 73). Parkinson dementia disease (PDD) and DLB have been described (74). More rarely, prominent language dysfunction resembling primary progressive aphasia and frontotemporal dementia with behavioral dysregulation and speech-related problems have been reported (75). Olfactory dysfunction, RBD, and dopaminergic deficit at DATSCAN have been proposed as possible premotor signs after their identification in otherwise asymptomatic p.A53T carriers (71, 72).

Conversely, a late-onset Parkinsonism with tremor as a rather constant motor sign and cognitive impairment ranging from mild cognitive decline to frank dementia have been reported in p.H50Q carriers, all with English ancestry (76, 77).

Patients with the p.A30P mutation, found in a single German family, present a condition similar to iPD, with onset around 60 years, incomplete penetrance, and a benign course of disease. Cognitive impairment and hallucination occur, although inconsistently (78, 79).

More severe phenotypes are related to the p. E46K and p.G51D variants. The first one, identified in a Basque family, causes a high penetrant and severe Parkinsonism, presenting at 50–65 years (80). Dementia with LB phenotype and autonomic dysregulation occur a few years after the onset of motor signs. However, disease severity may vary among families. Marked cardiac denervation has been found in patients and in p.E46K asymptomatic carriers (81).

Conversely, the Parkinsonian condition related to p.G51D, found in a few European and Asian cases, strongly differs from the late-onset PD caused by the other *SNCA* damaging missense mutations. The age of onset is very early, before 20 years in one case, and pyramidal signs, myoclonus and seizures coexist, inconstantly complicated by psychiatric symptoms, dementia, and autonomic dysfunction (82, 83).

Another early onset *SNCA*-related Parkinsonism, with no atypical signs, has been described in a Finnish family with the p.A53E *SNCA* mutation (84).

A proper definition of phenotypes related to *SNCA* pathogenic missense variants is limited by the extreme rarity of such mutations and by the lack of thorough clinical evaluations for each individual case. A comprehensive international database considering complete and standardized information from individual patients would help to overcome these limits.

Pathogenic *SNCA* variants are localized in exons 2 and 3, which encode for an α-helical domain with lipid binding activity and for a hydrophobic domain (85). These mutations probably prolong α-syn half-life by interfering with lysosomal degradation (86). The tendency of some of these variants to accelerate α-syn aggregation and to recruit tau proteins into inclusions has been demonstrated with *in vitro* and *in vivo* studies. Further investigation will clarify if phenotype differences for distinct missense mutations depend on toxic gain-of-function mechanisms or another prolonged mutated protein half-life (87).

Soon after the discovery of the role of *SNCA* in AD PD, common variants at this locus were investigated for association with sporadic PD. A positive association emerged between iPD and expanded alleles at the *NACP*-Rep1 repeat, located 10 kb upstream of the transcriptional start site of *SNCA* (88, 89). Many additional GWAS analyses since then have supported a statistically significant association between the risk for PD and several single-nucleotide polymorphisms (SNP) located both at the 5′ end and the 3′ end of the *SNCA* gene (46). Although the effect of each SNP is individually low (odd ratio 1.3), the cumulative risk can be substantial. The *NACP*-Rep1 alleles and the rs356168 SNP increase α-syn expression both *in vitro* and *in vivo*, supporting the hypothesis that common *SNCA* variants increasing α-syn expression also increase the risk for apparently sporadic PD (87).

#### AD Genes Awaiting Confirmation

In addition to these AD PD genes, many others have been proposed as monogenic causes of hereditary iPD-like conditions. However, their pathogenicity is still debated or waiting for confirmation. *GIGYF2* (GRB10-interacting GYF protein 2, MIM^*^612003; PARK11, MIM#607688), *HTRA2* (HTRA serine peptidase 2, MIM^*^610297; PARK13, MIM#606441), *UCHL1* (ubiquitin carboxyl-terminal esterase L1, MIM^*^191342; PARK15, MIM#613643), and *EIF4G1* (eukaryotic translation initiation factor 4-G, MIM^*^614251; PARK18, MIM#614251) variants were detected in families with late-onset Parkinsonism resembling iPD and segregating with autosomal dominant fashion. However, many studies denied their pathogenic role in PD and none of them is still considered as a PD gene (90, 91). With the advent of NGS and the increasing availability of whole exome/genome sequencing (WES/WGS) many genes such as *DNAJC13, CHCHD2, TMEM230, LRP10*, and *RIC3* have been identified in AD PD families. Variants in *CHCHD2* (coiled-coil-helix domain containing protein 2, MIM^*^616244), a gene involved in the mitochondrial respiratory function, were identified in a few families with AD PD (PARK22, MIM#616710). Patients presented variable disease onset (mean age 52 years), good response to levodopa, depression, and the absence of cognitive impairment. A brain autopsy of a *CHCHD2* PD patient revealed widespread LB pathology with amyloid plaques and neurofibrillary tangles in the brainstem, limbic regions, and cortex (92–94). Despite these results, large-scale studies did not support the causative role of *CHCHD2* in PD [reviewed in (95)]. A definitive confirmation of its pathogenicity is still lacking.

Evidence is less robust for the remaining four genes. The c.2564A>G mutation in *DNAJC13* (DNAJ/HSP40 homolog subfamily C, member 13, MIM^*^614334) gene was identified in patients with late onset (mean age 63 years) and slow progressive PD from a large Mennonite kindred. Brain pathology of three mutation carriers showed LB with cell loss in Meynert nucleus and SN, as well as tau pathology. However, no other PD cases with pathogenic variants in *DNAJC13* have been detected to date and its role in PD etiology has been recently reconsidered (91). An independent study in the same Mennonite kindred identified the c.422G>T variant in *TMEM230* (Transmembrane protein 230, MIM^*^617019) as the cause of the Parkinsonism segregating in that family. The same variant was also detected in a few sporadic PD cases (96). As no other rare pathogenic mutation in *TMEM230* has been detected in familial and sporadic PD patients, pathogenicity for this gene still needs confirmation (97).

Recently, the c.169C>A mutation in *RIC3* (resistance to inhibitors of cholinesterase 3, MIM^*^610509) was found to segregate with a variable onset (30–68 years) Parkinsonism in a large family from South India. Nine mutated patients across three consecutive generations presented typical PD with RBD, depression, and restless leg syndrome (98). *RIC3*, not detected in other PD cases, is also waiting for confirmation (99). Heterozygous mutations in *LRP10* (low-density lipoprotein receptor-related protein 10, MIM^*^609921) were detected in Italian kindred with late-onset PD and in other unrelated patients with Parkinsonism and dementia PDD and DLB (100). However, inconsistent findings emerged by replications studies worldwide, with no differences in *LRP10* variant frequency between patients and controls, and for the incongruity of segregation analysis (101). Other population and functional studies are required to elucidate the role of this gene in PD.

### Other Disease-Causing Genes Associated With PD

#### Other Movement Disorder Genes Possibly Manifesting as AD PD

An iPD phenotype can be related to genes known to cause other movement disorders. In particular, pathogenic variants in *GCH1* (GTP cyclohydrolase I, MIM^*^600225), the most common cause of levodopa-responsive dystonia (MIM#128230), have been found in patients and families with AD PD characterized by variable disease onset (mean age 43 years), long-term motor complications, and non-motor signs such as cognitive impairment, sleep disorders, hyposmia, and autonomic dysfunction, without dystonia. Nigrostriatal degeneration in affected patients was also proved by a reduced tracer uptake in SPECT studies (102). The association between PD phenotype and *GCH1* pathogenic variants was confirmed by further studies (103, 104). Also, triplet expansion variants in *ATXN2* (MIM^*^601517), responsible for autosomal dominant cerebellar ataxia type 2 (SCA2, MIM#183090), may cause a form of typical PD with onset after 40 years, good response to levodopa therapy without cognitive impairment, and cerebellar signs. LB pathology and neuronal loss have been documented, in the absence of cerebellar atrophy. A CAG repeat expansion exceeding 33 is considered pathogenic. However, while in SCA2 phenotypes the mean of repeats is 43; in PD cases, repeats are lower in number (mean 36 ± 1) and with CAA interruptions (105, 106).

#### GBA

Heterozygous variants in the *GBA* (acid beta glucosidase, MIM^*^606463) gene are the most common genetic risk factor for PD (MIM#168600) worldwide. *GBA* encodes the β-glucocerebrosidase (GCase), a lysosomal enzyme that cleaves the glucosylceramide sphingolipid into glucose and ceramide. Biallelic mutations in *GBA* cause Gaucher's disease (GD), the most common AR lysosomal storage disease with a variable involvement of the central nervous system. About 300 different pathogenic *GBA* variants have been found, many of them resulting in a significant loss of GCase activity (107). Different mutations can lead to different phenotypes of GD. Variants are overall classified according to the GD subtype they are related to. Mutations causing the non-neuronopathic GD type 1 are defined as mild (e.g., the c.1226A>G, also known as N370S in the traditional nomenclature); those causing neuronopathic GD type 2 and 3 are classified as severe (e.g., c.1448T > C, L444P) (108).

The association between *GBA* variants and increased risk of Parkinsonism arose after clinical observations of higher incidence of PD among GD type 1 patients and their heterozygous parents. This suggestion was confirmed by a large, worldwide, multicenter association study, and by many other papers which demonstrated higher frequencies of the GBA variants in PD patients compared to healthy controls (109). The N370S and the L444P mutations are the two most common alleles worldwide, accounting for 70–80% of *GBA* variants associated with PD in some populations. In particular, the N370S is the most frequent among Ashkenazi Jews from eastern Europe whereas L444P is more common among non-Jew European descendants (110). Nevertheless, *GBA* mutations represent only a risk factor for PD. Population studies showed that a little more than 9% of *GBA* mutation carriers develop PD (111). Heterozygotes for a *GBA* mutation have a 5-fold increased risk of developing PD compared to the age-matched general population. For homozygotes, the risk increase is 10- to 20-fold (108). Pathogenic variants in *GBA* are detected in 8.5% of PD patients (112). However, carrier frequency varies across different ethnic groups, ranging from 10 to 31% in Ashkenazi Jews, 2.9–12% in non-Jewish Europeans, and 2.3% in Norwegians (113). PD cases with *GBA* mutations are usually similar to iPD. However, some peculiarities are emerging by large population studies comparing carrier and non-carrier PD phenotypes and clinical features related to mild and severe mutations. In heterozygous *GBA* PD, the first symptoms manifest 3–6 years earlier than in iPD, a rigid akinetic motor phenotype is more common, and the response to levodopa is good. However, at least in a subset of patients, the progression of the disease can be faster and therapeutic outcomes are often limited by earlier development of motor fluctuations and dyskinesias, as well as cognitive decline after DBS (114, 115). *GBA* mutation carriers are also more likely to manifest non-motor symptoms. Cognitive involvement is more frequent, and the risk of developing dementia is at least 3-fold higher than in iPD (116, 117). Non-motor symptoms, such as hallucinations, depression and anxiety, impulse control disorders, RBD, and autonomic dysfunctions, are also more common among *GBA* patients, and more frequent in severe mutation carriers. Similarly, motor complications, including dysphagia, dysarthria, and freezing of gait, are more frequent in *GBA* carriers (118).

Compared to milder variants, severe *GBA* mutations are associated with a higher risk of developing PD, younger onset, worse motor progression, more frequent cognitive involvement, and more complex non-motor phenotype (27, 119).

*GBA* PD patients have a distributed pattern of white matter abnormalities involving the interhemispheric, frontal cortico-cortical, and parahippocampal tracts, and no gray matter atrophy at structural MRI (120). Spectroscopic MRI shows a neurodegeneration pattern more pronounced in the putamen than in the midbrain (121). *GBA* mutation carriers also differ from iPD cases in PET studies, showing reduced cerebral blood flow in the parieto-occipital cortex and reduced nigrostriatal function. This resembles the pattern typically seen in DLB, especially in severe mutation carriers (119).

Mutations in *GBA* are also a significant risk factor for DLB, conferring a more than 8-fold increased risk of developing this condition compared to controls (122). The association between *GBA* pathogenic variants and other Parkinsonian conditions is instead less consistent. Correlations with PSP are still weak (123, 124), but recent studies demonstrated an association between *GBA* variants and MSA (125, 126).

The exact mechanism by which *GBA* mutations lead to PD is still unclear. It is well-known that GCase is part of the endo-lysosomal pathway, particularly crucial in many pathogenetic pathways leading to PD. Mutated GCase is not able to fold properly and accumulates in different cellular compartments of the dopaminergic neurons, causing a cell stress response, damage, and neuronal death. In addition, the entrapment of the beta GCase in the endoplasmic reticulum reduces enzyme levels in the cell, triggering α-syn accumulation (127). Intriguingly, LB pathology has been found in cortical areas of brains from 10 PD patients with Parkinsonism and from almost all *GBA*-related PD cases who underwent autopsy. Less is known about the distribution of neuronal loss or additional neuropathology (128).

At present, clinical trials assessing the safety and the efficacy of molecules aiding proper GCase folding, improving enzymatic activity, or reducing the GCase substrates accumulation are ongoing (108).

### Autosomal Recessive Early-Onset Parkinsonisms

Among AR parkinsonisms, forms caused by biallelic pathogenic variants in the *PRKN, PINK1*, and *DJ-1* genes are thus far considered pure forms of EOPD. Together, they account for ~13% of EOPD cases (73). *VPS13C* and *PTRHD1* are mutated in families with EOPD complicated by pyramidal signs and cognitive involvement. Other genes such as *APT13A2, PLA2G6, FBXO7*, and the more recently identified *DNAJC6, SYNJ1*, and *VPSC13* are usually related to younger onset Parkinsonism, complicated by atypical motor and non-motor phenotypes. Biallelic mutations in another gene, *PODXL*, recently have been detected in siblings with a juvenile form of pure PD. However, this gene is still waiting for confirmation.

#### Pure EOPD Forms

The *PRKN, PINK1*, and *DJ1* genes share similar PD phenotypes and the same cellular pathway. They are involved in mitochondrial homeostasis and mitophagy. Variants in these genes can impair mitochondrial function, leading to cellular stress and neurotoxicity. *PRKN* encodes the E3-ubiquitin ligase, involved in the proteasome pathway for damaged proteins degradation and in mitochondrial homeostasis. *PINK1* encodes a mitochondrial kinase involved in mitophagy, acting upstream of Parkin. *DJ-1* encodes for a protein involved in the antioxidant response that shares biochemical pathways with *PINK1* and Parkin (129–131).

##### PRKN

Biallelic pathogenic variants in *PRKN* (Parkin, MIM^*^602544), are the most common genetic cause of early-onset pure Parkinsonism (PARK2, MIM#602544) (132). First identified in a Japanese family with AR PD, *PRKN* variants currently account for 10–20% of PD with onset within 40 years (133). The occurrence of biallelic mutation is inversely related to the age of the disease onset: the younger the onset, the higher the probability to detect homozygous or compound heterozygous *PRKN* carriers. The onset is usually before 40 years, with a median age of 31 years for the first motor signs. Juvenile onset, with the first symptoms within 20 years, is described in 16% of PD patients with biallelic *PRKN* mutations (73). Cardinal signs of *PRKN*-related PD are a more common bradykinetic motor phenotype, benign and slow progression, and good response to levodopa therapy, although frequently complicated by iatrogenic dyskinesia, to anticholinergic medication, and to DBS (26, 73, 134). Hyperreflexia and/or dystonia may occur, and lower-limb dystonia can be a presenting sign. Cognitive decline is uncommon, being as frequent as in the general population, and dementia is extremely rare. Sense of smell is usually well-preserved and additional features, such as psychiatric manifestations and dysautonomia, are also rare (134, 135). Disease duration can reach 50 years. Later disease stages may be complicated by freezing of gait, postural deformities, and motor fluctuations (136).

Unlike iPD, women and men are equally affected and the loss of dopaminergic striatal innervation, revealed by 18F-DOPA PET/SPECT, is rather symmetric and slowly progressive (134).

Neuronal loss in pigmented nuclei of the brainstem is the prominent feature found in brains of *PRKN* PD patients. Neurodegeneration is more prominent in the SNpc than in the *locus coeruleus*. Typical LBs containing α-syn have been identified in very few affected individuals (136, 137). Pathogenic variants are highly diverse, including exonic deletions or multiplications and missense, nonsense, and frameshift variants, described in homozygous or compound heterozygous states. Exonic rearrangements are the most common anomalies. Functional studies demonstrated protein loss-of-function or absence of protein due to nonsense mRNA decay for most of them (138, 139).

##### PINK1

The phenotype related to biallelic pathogenic variants in *PINK1* (PTEN-induced putative kinase 1, MIM^*^608309; PARK6 MIM#605909) is similar to *PRKN* PD, with early onset, good response to levodopa, and rare cognitive compromission. However, hyperreflexia is less common and hyposmia, dysautonomic features, and psychiatric symptoms including anxiety, psychosis, and affective disorders may occur (73). Neuronal loss is prevalent in SN and, contrary to *PRKN*-related disease, LBs have been found on neuropathological examinations (140).

*PINK1* biallelic mutations are the second most common cause of EOPD worldwide, accounting for 1–5% of cases, but with variations among ethnic groups, being more frequent in north Africa (90). More than 60 variants, including deletions and missense and nonsense variations, have been reported (73).

##### DJ-1

Biallelic variants in *DJ-1* (oncogene Dj1, MIM^*^602533) cause a rare Parkinsonism (PARK7, MIM#606324), the third most common AR PD after the *PRKN-* and *PINK1-*related forms, accounting for the 0.4 and 1% of EOPD cases, respectively (1). Patients usually share the same phenotype of both *PRKN* and *PINK1* cases, presenting early onset slowly progressive Parkinsonism (mean age 27 years), good response to dopaminergic therapy, frequent focal dystonia, motor complications upon treatment, and psychiatric symptoms, in particular anxiety that often presents as the first symptom (73, 132). Unlike *PRKN* and *PINK1*, additional features, including depression, cognitive decline, motor neuron disease, and bulbar signs are seldom described (141, 142). The only reported neuropathological brain examination showed loss of neurons in the substantia nigra and locus coeruleus with widespread α-syn, akin to sporadic PD brains (142).

##### Heterozygous Variants in *PRKN, PINK1*, and *DJ-1*

While biallelic pathogenic variants in *PRKN, PINK1*, and *DJ-1* are clearly causative for EOPD, the role of single heterozygous mutations in these genes is debated. *PRKN* and *PINK1* heterozygous mutations have been detected in a substantial number of PD patients but also in healthy controls, raising the question of whether they may contribute to the disease or are incidental findings. The results of many case-controlled studies suggest that they may represent minor susceptibility factors that mildly contribute to the risk of sporadic PD (Parkin odds ratio 2:53; *PINK1* odds ratio 1:65) (143, 144). Interestingly, multimodal neuroimaging and electrophysiologic studies disclosed a latent nigrostriatal impairment in otherwise healthy subjects carrying heterozygous Parkin or *PINK1* mutations (145–147). However, the role of heterozygous variants in these AR genes cannot be conclusively established as prospective studies of healthy heterozygous carriers are still lacking (144).

#### Atypical EOPD Forms

##### VPS13C

*VPS13C* (vacuolar protein sorting 13 homolog C, MIM^*^ 608879) is the most recently identified gene causing a rare, atypical form of early-onset Parkinsonism (PARK 23, MIM#616840). To date, only three families have been described. All affected patients presented asymmetric akinetic-rigid Parkinsonism starting from age 25 to 45, with initially good response to dopaminergic treatment. The disease progression is severe and rapid with dramatic early cognitive involvement, dysautonomia, limb dystonia, hyperreflexia, and pyramidal signs leading to tetraplegia. The patients are bedridden after about 10 years of disease. MRI documented asymmetric brain atrophy. Neuropathology of a single case showed widespread and abundant α-syn positive LBs and neuritis, together with tau pathology with neurofibrillary tangles (148). *VPS13C* encodes the vacuolar protein sorting 13 protein involved in mitochondrial activity and vesicular trafficking. It has been shown that *VPS13C* mutations alter mitochondrial function and *PINK1*-Parkin-dependent mitophagy (149).

##### PTRHD1

Homozygous missense mutations and 28-nucleotid frameshift deletion in *PTRHD1* (peptidyl-tRNA hydrolase domain-containing 1, MIM^*^617342) have been recently identified in two unrelated consanguineous Iranian families and in a sub-Saharan African kindred with early onset Parkinsonism and intellectual disability (150, 151). Motor signs of Parkinsonism appeared, at 20–30 years of age. Phenotypes were variably complicated by muscle stiffness, postural tremor, pyramidal signs, sensory-motor polyneuropathy, behavioral disorders, and hypersomnia. No other *PTRHD1* cases have been identified to date (152). Chromosomal microdeletions encompassing the *PTRHD1* gene have been previously related to many syndromes with intellectual disability. *PDRHT1* encodes a protein involved in the ubiquitin proteasome pathway. Intriguingly, the pathogenic variants causing Parkinsonism are in the ubiquitin-like (UBL) domain-binding site of the protein. The suppression of the ubiquitin protein degradation is a well-known mechanism of PD. Despite these findings, further population and functional studies are needed to confirm the role of this rare gene in determining PD.

### Juvenile Parkinsonism

Juvenile Parkinsonism includes those forms of PD with onset within 21 years, often combined with other hyperkinetic movement disorders and neurological and imaging abnormalities (153). With the exclusion of *PRKN* mutations, responsible for 77% of juvenile PD (see section Other Movement Disorder Genes Possibly Manifesting as AD PD) and the extremely rare *PODXL*-related cases, more than 90% of patients have a complex or atypical presentation, with dystonia, pyramidal signs, neuropsychiatric disorders, abnormal eye movements, and brain imaging. Many genes have been associated with AR young JOPD. This section will focus on those forms in which Parkinsonism is the predominant sign.

#### Pure JOPD

##### PODXL

A homozygous frameshift variant in the *PODXL* gene (podocalyxin-like, MIM^*^602632) has been described in three siblings from an Indian consanguineous family, who developed a pure levodopa-responsive Parkinsonism manifested at 13–17 years of age, later complicated by dyskinesia and off-dystonia, with no additional signs. *PODXL* encodes a glycoprotein involved in the regulation of neurite outgrowth. The frameshift mutation (c.89_90insGTCGCCCC) resulted in a complete loss of protein function (154). Replications of these findings and confirmation of *PODXL* as a causative PD gene are still awaited.

#### Atypical

##### JOPD

*DNAJC6* Biallelic damaging variants in *DNAJC6* (DNAJ/HSP40 homolog, subfamily c, member 6, MIM^*^ 608375) are associated with a form of juvenile Parkinsonism (PARK19, MIM#615528), with a mean age of onset at 11 years (range 7–42 years). The wide phenotypic spectrum ranges from typical pallido-pyramidal syndrome to pure EOPD. Motor signs at onset vary from tremor to bradykinesia but the disease later manifests with Parkinsonism, postural instability, and usually good response to levodopa, often limited by treatment-induced dyskinesia, psychosis, and hallucinations (155, 156). In complex forms, Parkinsonism is often complicated by atypical features such as cognitive decline, spasticity/pyramidal signs, dysarthria/anarthria, seizures, and hallucinations. Disease progression is severe, especially in cases with younger onset, who need a wheelchair after 2–10 years of disease (155, 157, 158). Brain atrophy has been described in all but one case (158). Conversely, those cases with pure Parkinsonism show variable age of onset, slow disease progression, and good response to dopaminergic therapies (156). *DNAJC6* encodes for Auxilin, a protein involved in calthrin-mediated endocytosis. Mutations in *DNAJC6* cause an impairment in synaptic vesicle recycling, compromising endocytosis (155). A clear genotype-phenotype correlation has been defined: nonsense mutations have been related to juvenile complex Parkinsonism, while patients with missense mutations or variants resulting in reduced protein production have been found in pure Parkinsonism cases (156, 157).

##### SYNJ1

*SYNJ1* (synaptojanin, MIM^*^604297) mutations cause another AR form of JOPD (PARK20, MIM#615530) characterized by motor PD features presenting at a median age of 21 years (range 12–31 years), poor response to levodopa treatment, early induced dyskinesias, gait disorders, and dysarthria/anarthria (159, 160). In most cases, dystonia, cognitive decline, seizures, and oculomotor abnormalities were described as well (33). Cerebral cortical atrophy, quadrigeminal plate thinning, and hippocampal T2-hyperintensity at MRI have been inconsistently reported (95). Recently, a good response to clonazepam therapy, especially for trunk dystonia, has been reported in two *SYNJ1* compound heterozygous siblings with diplopia, dystonia, and Parkinsonism (161). At present, 12 families with atypical Parkinsonism and biallelic *SYNJ1* variants have been described (33, 161). *SYNJ1* encodes for a poly-phosphoinositide phosphatase, which contains two consecutive phosphatase domains. Mutations affecting the SAC1-like domain are responsible for Parkinsonism while variants impairing the dual phosphatase domain cause a recessive infantile epilepsy syndrome with severe and progressive neurodegeneration (MIM# 617389) (162). No pathology from *SYNJ1* patients with Parkinsonism is available today. However, brain neuropathology on a child with biallelic *SYNJ1* pathogenic variants affected by refractory epilepsy and severe neurodegeneration showed white matter atrophy and prominent cell loss, tau pathology, and neurofibrillary tangles in SN and, although less represented, in basal ganglia (163).

##### ATP13A2

*ATP13A2* (ATPase 13A2, MIM^*^610513) causes Kufor Rakeb syndrome (MIM#606693), an AR atypical JOPD with onset before 20 years (164, 165). The disease is characterized by a combination of partially levodopa-responsive Parkinsonism and pyramidal signs, complicated in most cases by dementia, hallucinations, dystonia, impaired saccadic movements, vertical gaze palsy, and mini-myoclonus. Symptoms at onset are variable, encompassing akineto-rigid syndrome, learning disability, cognitive deterioration during school years, or behavioral dysfunctions. Progression of the disease is slow, sometimes associated with cerebellar dysfunction (33). Brain MRI shows diffuse atrophy and, in many cases, iron accumulation in the putamen and caudate (165, 166). *ATP13A2* encodes for a lysosomal P5-type ATPase protein that transports inorganic cations and regulates endo-lysosomal cargo sorting and neuronal integrity. Homozygous or compound heterozygous variants in *ATP13A2* also have been associated with complex hereditary spastic paraplegia (SPG78, MIM# 617225), with no Parkinsonism in most reported cases and to neuronal ceroid lipofuscinosis. In brains from Kufor Rakeb patients, neuronal and glial lipofuscin deposits at the cortex, cerebellum, and basal ganglia have been described (167, 168).

##### PLA2G6

Biallelic mutations in *PLA2G6* (phospholipase A2, group 6, MIM^*^603604) cause an early-onset dystonia-Parkinsonism (PARK14, MIM#612953) with variable age at onset (10–30 years) (169, 170). The disease is also characterized by rapid cognitive decline, pyramidal signs, eye movement abnormalities, psychiatric and behavioral problems, cerebellar ataxia, and autonomic dysfunction. The response to levodopa therapy is good but compromised by early-onset treatment-induced dyskinesias. The progression of the disease is rapid and severe, leading to loss of autonomy (171). MRI may show brain iron accumulations and frontal lobe and general white matter atrophy (172). Beyond atypical Parkinsonism, biallelic pathogenic variants in *PLA2G6* are also the cause of other neurodegenerative diseases, including infantile neuroaxonal dystrophy (INAD, MIM#256600) and idiopathic neurodegeneration with brain iron accumulation, type 2 (NBIA2, MIM# 610217), all sharing many pathological and clinical features. They are characterized by spheroid axonal inclusions in the brain and motor regression, progressive cognitive decline, axial hypotonia, spasticity, bulbar and ophthalmic dysfunctions, dystonia, and cerebellar atrophy, starting in the first year of life (171). *PLA2G6* encodes for calcium-independent phospholipase A2 beta enzyme (iPLA_2_β), which participates in cell membrane homeostasis, mitochondrial function, fatty acid oxidation, and calcium signaling. Defects of this protein lead to membrane fluidity alteration and neuronal function impairment. a-syn with LBs, Lewy neurites, and neuroaxonal dystrophy are documented in patients with PARK14 and in INAD cases. Differences in phenotypes are related to the effects of the mutations: variants causing loss of *PLA2G6* catalytic activity leads to INAD/NBIA2, whereas mutations altering substrate specificity or regulatory domains are responsible for PARK14 (173).

##### FBXO7

A form of juvenile pallido-pyramidal syndrome (PARK15, MIM#260300) is caused by biallelic mutations in *FBXO7* (F-box only protein, MIM^*^605648). Parkinsonism is usually the first manifestation with rigidity, bradykinesia, postural instability, and, less frequently, tremor, around the age of 17 (range 10–52) years. Pyramidal signs are common and cognitive decline, vertical gaze palsy, and autonomic dysfunctions also occur, although less frequently. Response to levodopa is good but often limited by psychiatric and dyskinetic complications. No abnormalities have been detected in brain MRI (174, 175).

*FBXO7* encodes an adaptor protein involved in substrate degradation and in mitochondrial maintenance interacting with *PINK1* and Parkin.

### X-Linked PD

Pathogenic whole gene deletion, missense, and splicing mutations in *RAB39B* (RAS-associated protein RAB39B, MIM^*^ 300774) were identified in a few families with non-progressive intellectual disability, macrocephaly, and early-onset Parkinsonism in males (Waisman syndrome, MIM# 311510). Tremor was the presenting motor sign, followed by a levodopa-responsive akinetic-rigid Parkinsonism. Seizures were inconsistently present. Brain atrophy of one affected patient documented a clear α-syn with LBs in the SN and in the cortex, together with tau positive neurofibrillary tangles in the SN and axonal spheroid in the white matter of basal ganglia (176–178). Other mutations of *RAB39B* were detected in patients with intellectual disability of variable degree, autism, seizure, and macrocephaly without PD (95), as well as in male and female patients with early- or later-onset Parkinsonism, respectively, without intellectual disability (179). *RAB39B* encodes a neuronal protein involved in vesicular recycling trafficking and in the maintenance of α-syn homeostasis. Parkinsonian phenotypes have been mainly related to loss of function mutations (179). To date, *RAB39B* is a proven but rare cause of Parkinsonism and intellectual disability in males. Many studies failed to identify pathogenic alterations of this gene in large cohorts of PD patients (180).

## Discussion

The advent of next generation sequencing (NGS) increased the number of genes known to cause Mendelian forms of Parkinsonism. They are involved in many specific biological pathways, suggesting that several cellular functions are critical in the pathogenesis of PD. *SNCA* is directly responsible for altering the expression of α-syn, the main component of LBs (181, 182). *PRKN, PINK1*, and *DJ-1* are related to mitochondrial function and mitophagy (183–185), as well as other genes linked to classical and atypical PD such as *FBXO7, PLA2G6, VPS13C*, and *CHCHD2*, which are involved in the mitochondrial quality control system (186). The impairment of lysosomal function has also been linked to the pathogenesis of PD. Altered *LRRK2* compromises cell autophagy, reducing α-syn degradation (187, 188), *ATP13A2* mutations cause lysosomal dysfunctions (189), and reduced enzymatic activity of GCAse determinates α-syn accumulation, endoplasmic reticulum stress, and mitochondrial dysfunction (190). Recently, a novel disease mechanism involving vesicular trafficking and synaptic endocytosis has been proposed after the identification of many other PD-related genes, such as *VPS35, DNAJC6, SYNJ1*, and *LRP10* (49).

A precise genetic diagnosis enables proper genetic counseling according to the mode of inheritance and penetrance of the mutation and may help define the disease prognosis and influence therapeutic choices.

**Figure 1:**
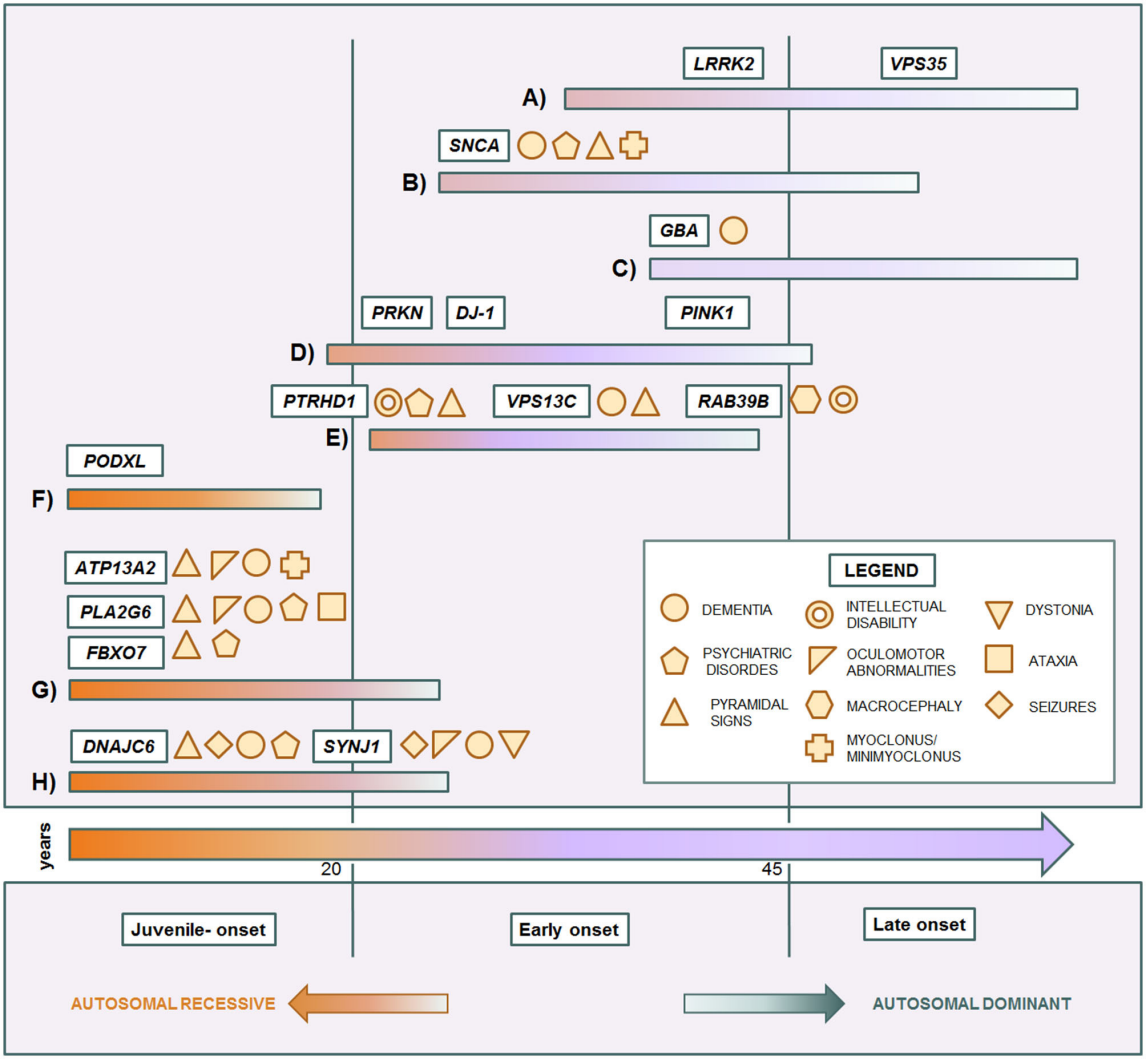
Genotype-phenotype correlation in monogenic Parkinsonian conditions. Central arrow: time of onset. Each colored bar represents a subset of conditions. **(A)** iPD-like, late-onset autosomal dominant Parkinsonisms. **(B)** Complicated autosomal dominant Parkinsonisms. **(C)** Genetic risk factor causing late-onset Parkinsonism. **(D)** Typical autosomal recessive early-onset Parkinsonisms. **(E)** Complicated autosomal recessive early onset Parkinsonisms. **(F)** Juvenile uncomplicated Parkinsonism. **(G)** Juvenile pallido-pyramidal syndromes. **(H)** Juvenile atypical Parkinsonisms.

Some genes and some mutations are associated with specific phenotypes. The relationship between clinical phenotypes and their molecular bases is depicted in Figure 1. The *LRRK2* p.G2019S mutation usually causes a slow progressive iPD-like condition with variable age of onset. *SNCA* pathogenic variants are responsible for a more aggressive Parkinsonism with cognitive decline and other non-motor features. Instead, *GBA* variant carriers can present more heterogeneous phenotypes, ranging from absence of disease to severe Parkinsonian conditions. In affected carriers, the disease usually manifests as classical late-onset levodopa-responsive PD. However, in a subgroup of patients, the condition can be more severe, sometimes presenting with cognitive decline as PDD or DLB (122, 191). Among EOPD cases, *PRKN, PINK1*, and *DJ-1*, sharing mitochondria and mitophagy related functions, are usually responsible for pure PD forms with only motor signs, slow progression, and good response to dopaminergic therapy. Complex EOPD forms with rapid progression, early cognitive deterioration, and additional movement disorders instead are usually related to new AR genes. Except for *PODXL*, mutations in genes causing juvenile Parkinsonisms are always related to complex phenotypes in which pallido-pyramidal signs, oculomotor abnormalities, cognitive impairment, and seizures variably occur. Intellectual disability in males is a red flag for *RAB39B* mutations. However, phenotypes rarely overlap, hindering the achievement of the correct diagnostic assumptions. Early onset *LRRK2*-related PD as well as milder forms of *DNAJC6*-related Parkinsonism may present with a phenotype resembling pure *PRKN/PINK1* recessive cases (132, 156). Clinical features in *SNCA* triplication carriers are similar to the *VPS13C-*related phenotype (63, 148), while the complex phenotype of *SNCA* p.G51D patients, characterized by precocious disease onset, pyramidal signs, myoclonus, seizures, and, inconstantly, cognitive decline, significantly overlaps with *DNAJC6-* and *SYNJ1*-related phenotypes (82, 155, 157–160). Differential diagnosis among complex juvenile Parkinsonisms is more tangled and only partially helped by ancillary tests (33). The easier availability of NGS in clinical practice has notably unraveled the emerging genotype and phenotype heterogeneity in Parkinsonian conditions. However, the clinical reasoning is too often overshadowed by excessive reliance on the diagnostic power of this technique. Exonic, multiexonic, and/or whole gene rearrangements, frequently implied in many PD genes, including *PRKN, PINK1, DJ-1*, and S*NCA*, and even pathogenic repeat expansions of *ATXN2*, are not generally identified by NGS techniques. The request of the proper molecular analysis, and thus the possibility to thereby reach the correct diagnosis still requires a scrupulous clinical observation and, whenever possible, a focused clinical diagnostic suspicion, within the frame of accurate genetic counseling.

Precision medicine is becoming a reality as well for PD. The presence of defined pathogenic mechanisms, identifiable with genetic testing, is appealing for targeted therapies. Ongoing clinical trials are mainly recruiting participants carrying mutations in specific genes. However, some of these therapies might also be used for broader iPD cohorts. GCse, α-syn, *LRRK2*, and mitochondrial functions are currently seen as targets for personalized treatments.

The reduction of α-syn accumulation has been the first target of precision medicine. Many different therapeutic approaches have been proposed and even investigated in clinical trials.

One of them aims to reduce the synthesis of α-syn before its aggregation by neutralizing mRNA molecules with RNA interference (RNAi) technologies (192, 193), others by reducing *SNCA* transcription with molecules that interfere with histones acetylation (2-adrenergic receptor agonists) (194), or by using antisense oligonucleotide (ASO) therapy (49). A second therapeutic approach, aimed at preventing *SNCA* misfolding or aggregation, utilizes small antibody fragments (intrabodies) that prevent oligomerization by binding intracellular α-syn (195). Two such molecules are currently in early clinical trials (196, 197). An alternative strategy is the enhancement of α-syn clearance by immunotherapies or the activation of autophagy. The first method is based on passive immunization with α-syn specific antibodies or active immunization with injections of modified α-syn stimulating the production of endogenous antibodies. The second one is based on the administration of autophagy enhancers such as rapamycin, lithium, or the antineoplastic drug nilotinib (198). Clinical trials are ongoing for these compounds as well.

Another target of great interest is GCase. Clinical trials for *GBA*-targeted therapies are studying drugs able to increase GCase production or activity. The glucosylceramide synthase inhibitor GZ/SAR402671, which reduces glucocerebrosidase substrates, and ambroxol hydrochloride, a small chaperone molecule able to increase GCase activity, are present research targets. Other small molecular chaperones have been shown to reduce α-syn accumulation in GBA PD patients and for one of them a clinical trial is currently running (Netherlands Trial Register: NTR6960). Another way to restore GCase activity is to introduce wild-type *GBA* genes into the genome of *GBA* mutation carriers. Gene therapy, at present under investigation in other neuromuscular diseases, also will be tested in *GBA-*mutated patients (49, 199).

The evidence that *LRRK2* kinase activity inhibition reverses the pathological features and reduces α-syn accumulation in cellular and animal models makes this protein another candidate for a target therapy. Preclinical research investigated many *LRRK2* inhibitors that failed in brain penetration or in pharmacokinetic properties. Recently, two molecules with exceptional potency and selectivity in inhibiting *LRRK2* kinase activity and a good safety profile have been identified (200–202). Based on the good results on animal models, the administration of this molecule started in healthy and affected human subjects, with and without *LRRK2* mutations (ClinicalTrials.gov Identifier: NCT03710707; ClinicalTrials.gov Identifier: NCT04056689).

Finally, various therapeutic approaches focusing on mitochondrial dysfunctions have been proposed. Molecules improving mitochondrial functions, such as kinetin triphosphate, able to ameliorate the kinase activity of mutated *PINK1*, and selective MAO-B inhibitors, including selegilin and regasilin, are being studied. Good results of these treatments have been reported only after a proper stratification of patients, with the administration of these compounds in the “mitochondrial subtypes PDs” (203).

Currently, genetically determined PD offers the unique framework of a constant dialogue between cell biology, clinical acumen, medical genetics, and targeted therapy. Only appropriate genetic testing allows identification of the specific condition in patients, pointing which pathway, or pathways, is involved in the underlying pathogenesis for that case. Most of the ongoing trials about target therapy involve PD cases with molecular diagnosis as positive controls for the different forms of PD. In the era of precision medicine, the importance of genetics in PD is overstepping the boundaries of the research to play an increasingly pivotal role in clinical practice. Nevertheless, the identification of the compromised pathways and genes, driving the choice of the proper therapy, is still strictly dependent on clinical reasoning that, despite the availability of innovative diagnostic techniques, continues to be the irreplaceable key in reaching a genetic diagnosis.

## Author Contributions

DG: conceptualization, investigation, data curation, original draft, and visualization. MP: data curation, methodology, supervision, review, and editing. MT: resources, methodology, supervision, review, and editing. AP: methodology, data curation, supervision, review, and editing. SP: conceptualization, investigation, data curation, original draft, methodology, and supervision. All authors contributed to the article and approved the submitted version.

## Funding

This research received no external funding. AP was funded by Sapienza, University of Rome.

## Conflict of Interest

The authors declare that the research was conducted in the absence of any commercial or financial relationships that could be construed as a potential conflict of interest.

## Publisher's Note

All claims expressed in this article are solely those of the authors and do not necessarily represent those of their affiliated organizations, or those of the publisher, the editors and the reviewers. Any product that may be evaluated in this article, or claim that may be made by its manufacturer, is not guaranteed or endorsed by the publisher.
